# PReS-FINAL-2186: Monoallelic mutations of familial hlh-related genes associated to macrophage activation syndrome

**DOI:** 10.1186/1546-0096-11-S2-O21

**Published:** 2013-12-05

**Authors:** C Bracaglia, E Sieni, V Cetica, M Da Ros, A Insalaco, C De Fusco, C Micalizzi, F De Benedetti, M Aricò

**Affiliations:** 1 Division of Rheumatology, Department of Pediatric Medicine, IRCCS Ospedale Pediatrico Bambino Gesù, Rome, Italy; 2 Department of Pediatric Hematology-Oncology, Meyer Children's Hospital, Florence, Italy; 3 Department of Pediatric Hematology and Oncology, Pausillipon Children's Hospital, Naples, Italy; 4 Department of Pediatric Hematology-Oncology, G. Gaslini Children's Hospital, Genoa, Italy

## Introduction

Macrophage Activation Syndrome (MAS) is a severe complication of rheumatic diseases, frequently associated with systemic juvenile idiopathic arthritis (sJIA), but also described in others pediatric inflammatory disorders including Juvenile Systemic Lupus Erythematosus (SLE) and Kawasaki disease. Due to the close resemblance to a group of histiocytic disorders collectively known as hemophagocytic lymphohistiocytosis (HLH) it is currently classified among the secondary or acquired forms of HLH, and the term *rheu-HLH *has been proposed.

## Objectives

To describe clinical, functional and genetic features of MAS in the Italian HLH registry.

## Methods

We reviewed the HLH Italian National Registry to select patients with MAS defined according to the diagnostic criteria established by the Histiocyte Society (so called HLH criteria) with confirmed diagnosis of rheumatologic or autoimmune disease. Clinical data were collected. Functional screening of perforin expression and degranulation was performed using flow-citometry. Genetic study was carried out by direct sequencing of currently known genes that cause familial HLH.

## Results

Among the 813 patients referred to the Registry, 38 (5%) were diagnosed as MAS in the context of a rheumatic disease. Of them 13 were male and 25 female; 30 Caucasian and 8 Indian. Median age was 94 (quartiles: 37; 94; 136; 708) months. The underlying diseases were: sJIA (n = 28), SLE (n = 4), Kawasaki disease (n = 1), Juvenile Dermatomyositis (n = 1), undefined rheumatologic (n = 3) or autoimmune (n = 1) disease. The clinical **features included**: fever (n = 28/28, 100%), splenomegaly (n = 17/28, 61%), neurologic manifestations (n = 7/28, 25%), anemia (n = 15/25, 60%), thrombocytopenia (n = 14/25, 56%), neutropenia (n = 3/25, 12%), hypertriglyceridemia (n = 14/25, 56%), hypofibrinogenemia (n = 7/25, 28%), hyperferritinemia (n = 22/23, 96%; quartiles: 2.430, 10.264, 15.953, 96.000 ng/ml), hemophagocytosis (n = 9/25, 36%). Four patients (10.5%) died for disease progression. Functional screening of FHL was performed in 22 cases: perforin expression was normal in 14 and reduced in 8 (36%); degranulation was defective in 3/18 (17%) and normal in the remaining 15/18 cases. At least one test was defective in 10/23 (43%). Mutation analysis included PRF1 (n = 36), UNC13d (n = 22), STX11 (n = 33) and STXBP2 (n = 19) and allowed to identify monoallelic mutations in 11 of the 38 patients (29%), as follows: PRF1 (n = 8/36, 22%), STX11 (1/33, 3%), STXBP2 (3/19, 16%).

## Conclusion

A significant portion of patients with MAS appear to have functionally defective cytotoxic activity and carry monoallelic variants in genes associated with familial HLH. These data suggest additional similarities in genetic background and cytotoxic activity abnormalities between MAS associated with rheumatic diseases and familial HLH.

## Disclosure of interest

None declared.

